# The Role of Temperament Traits in Bipolar Disorder: Neuroimaging Study

**DOI:** 10.1155/da/5974860

**Published:** 2025-05-10

**Authors:** Kirill Markin, Artem Trufanov, Dmitriy Tarumov, Alexander Krasichkov, Yulia Shichkina, Mikhail Kupriyanov

**Affiliations:** ^1^Psychiatry Department, Military Medical Academy, Saint Petersburg, Russia; ^2^Department of Neurology and Manual Medicine of the Faculty of Postgraduate Education, Pavlov First Saint Petersburg State Medical University, Saint Petersburg, Russia; ^3^Department of Computer Science and Engineering, Saint-Petersburg Electrotechnical University “LETI”, Saint Petersburg, Russia; ^4^Radio Engineering Systems Department, Saint Petersburg Electrotechnical University “LETI”, Saint Petersburg, Russia

**Keywords:** bipolar disorder, functional connectivity, impulsivity, neuroimaging, temperament

## Abstract

**Purpose:** This study aimed to identify temperament traits alterations in bipolar disorder (BD) and explore their potential neuroimaging correlates using resting-state functional magnetic resonance imaging.

**Methods:** We assessed seed-to-voxel alterations in four large-scale brain networks (Salience, Frontoparietal, Default Mode, and SensoriMotor) in 49 patients with BD and 49 healthy individuals according to the difference of temperamental traits (Reward Dependence, Novelty Seeking, Harm Avoidance, and Persistence). Also, we measured the relationship of temperamental traits with the severity of manic and depressive symptoms and impulsivity.

**Results:** Lower Reward Dependence (t-Welch's (87.1) = −2.50; *p*=0.014) in bipolar patients was associated with increased functional connectivity between Salience Network and Default Mode and FrontoParietal Networks. Higher Novelty Seeking (t-Welch's (87.3) = 4.37; *p* < 0.001) was associated with increased functional connectivity within FrontoParietal Network, whereas its functional connectivity with Visual and Dorsal Attention Networks was decreased. Higher Harm Avoidance (t-Welch's (82.8) = 4.85; *p* < 0.001) was associated with increased functional connectivity between FrontoParietal Network and basal ganglia. Lower Persistence (*U* = 998; *p*=0.002) was associated with decreased functional connectivity within FrontoParietal Network and with Default Mode Network. Higher persistence in bipolar patients was associated with greater severity of manic symptoms (Spearman's rho = 0.302, *p*=0.018), while lower Reward Dependence was associated with increased severity of depressive symptoms (Pearson's *r* = −0.388, *p*=0.003). Harm Avoidance negatively correlates with Persistence (Pearson's *r* = −0.525, *p* < 0.001) and positively with reward dependence (Pearson's *r* = −0.259, *p*=0.036). We also found a negative correlation between impulsivity and Reward Dependence (Pearson's *r* = −0.312, *p*=0.029) and positive correlation between impulsivity and Novelty Seeking (Pearson's *r* = 0.525, *p* < 0.001).

**Conclusions:** The findings demonstrate a possible functional neuroimaging basis for altered temperamental traits in patients with bipolar disorder.

## 1. Introduction

Bipolar disorder (BD) is characterized by the presence of manic and depressive episodes in patients [1] and contributes significantly to disability among the working population, ranking 20th among all diseases [2]. It is a disorder with high genetic predisposition and heritability [3, 4], with clinical manifestations typically debuting at a young age [5]. Notably, the incidence rate in the 20–24 age group has gradually increased over the past 30 years, from 51.76 to 58.37 per 100,000 [6]. In the diagnostic paradigm of the prevailing categorical approach, the misdiagnosis rate for BD remains relatively high (ranging from 40% to 70%) [7, 8]. Differential diagnosis is often complicated by insufficient attention from clinicians and patients to hypomanic states, the higher presentation rates of depressive phases, or the misinterpretation of manic symptoms as psychotic features. Additionally, frequent comorbidity with other disorders [9] exacerbates these challenges. In at least one-third of cases, it takes 10–15 years from the initial manifestations to the diagnosis of BD [10].

Some contemporary scientific hypotheses propose a continuum [11], wherein BD and major depressive disorder manifest across three key domains of human functioning: activity, cognition, and emotion [12]. According to these proposed models, diagnostic difficulties may be mitigated by focusing on the severity of dysfunction within each domain [13]. In addition, there is growing evidence for the importance of transnosological symptoms in patients with affective disorders, such as anhedonia and impulsivity, as well as predisposing factors, which include specific personality traits [14–17].

Of particular importance in responding to external stressors is an individual's temperament, whose relationship with various aspects of health and mental disorders has been repeatedly demonstrated in population studies [18, 19]. Threre is also some evidence that altered temperament traits are associated with affective disorders [19–22]. Moreover, some of these changes are also found in relatives of patients with affective disorders [23, 24]. Numerous theoretical models of temperament traits have been developed, most of which emphasize a predisposition to developing affective-spectrum disorders [23]. One of the most well known and extensively studied models over the past 35 years is R. Cloninger's model [25]. This model is based on a biopsychosocial approach, linking personality traits to genetic predisposition, behavior, and neurotransmitter system functioning [26, 27].

At the same time, the search for biomarkers of affective disorders, particularly neuroimaging biomarkers, could facilitate addressing diagnostic challenges [28]. Systematizing the existing data from original studies suggests the identification of the most involved brain regions in the pathogenesis of affective disorders [29–32]. These findings are gradually being reflected in studies aimed at refining our understanding of pathogenesis [33], improving differential diagnosis [34, 35], identifying high-risk groups prior to disorder onset [36, 37], and determining the most appropriate psychopharmacotherapy [38, 39] using advanced magnetic resonance imaging (MRI) techniques.

Furthermore, neuroimaging has been actively employed to objectify temperamental traits, with the relative stability of neural network activity demonstrating a trait-like nature [40]. A large meta-analysis, encompassing data from over 3,000 respondents, revealed structural (in the parietal and temporal lobes) and functional (in the parietal, frontal, and temporal lobes) changes associated with varying degrees of Harm Avoidance in healthy individuals [41]. A similar original study conducted on 360 students enabled the development of a model to predict temperamental traits based on functional connectivity patterns in frontal-subcortical circuits [42]. Emerging studies with comparable designs are now being conducted on patients with mental disorders. For instance, an evaluation of changes in functional connectivity across different brain regions in stable outpatients with psychoses (including BD and schizophrenia) revealed correlations with specific personality features such as sensory hypersensitivity, negative emotional balance, impaired attentional control, avolition, and social mistrust [43]. Interestingly, character and temperament traits in patients compared to control groups accounted for the majority of differences in functional connectivity.

The aim of this study was to compare temperamental traits in patients with BD and healthy controls (HC) and potentially identify functional connectivity changes in large-scale brain networks associated with these differences. We hypothesized that patients with BD would exhibit distinct temperamental traits compared to healthy controls and that these differences, in turn, would be linked to alterations in the functional connectivity of key large-scale brain networks, specifically those most commonly affected in BD: the Default Mode Network (DMN), Salience Network (SN), FrontoParietal Network (FPN), and Sensorimotor Network.

## 2. Materials and Methods

This study was conducted using a shared neuroimaging dataset from the UCLA Consortium for Neuropsychiatric Phenomics, which included imaging and clinical data for 49 BD patients in various phases and 130 healthy controls (HC) (from which we selected 49 respondents matched for sex and age with the study group) [44]. According to the data listed in the dataset, the established diagnosis with last episode suggested patients with BD in the following states: 22 in full/partial remission (of which with last manic/hypomanic—9, depressive—13), 18 with current mild/moderate illness (of which with manic/hypomanic—6, mixed—6, depressive—6) and 9 in current severe episode (of which with manic/hypomanic—1, mixed—3, and depressive—5). Demographic: sex, age; psychometric: Hamilton Depression Rating Scale (HAMD) for depressive symptoms severity, Young Mania Rating Scale (YMRS) for manic symptoms severity, The Temperament and Character Inventory (TCI-125) for temperament traits, Barrat Impulsivity Scale (BIS11) for impulsivity severity; and structural and resting-state functional MRI data were extracted for all 98 respondents.

Neuroimaging data were acquired using a 3T Siemens Trio scanner. Functional MRI data were obtained with a T2*⁣*^*∗*^-weighted echoplanar imaging (EPI) sequence using the following parameters: slice thickness = 4 mm, 34 slices, TR = 2 s, TE = 30 ms, flip angle = 90°, matrix = 64 × 64, field of view (FOV) = 192 mm. High-resolution T1-weighted anatomical scans (MPRAGE) were acquired with parameters: slice thickness = 1 mm, 176 slices, TR = 1.9 s, TE = 2.26 ms, matrix = 256 × 256, FOV = 250 mm. Diffusion-weighted imaging data were obtained with the following parameters: slice thickness = 2 mm, 64 diffusion directions, TR/TE = 9000/93 ms, flip angle = 90°, matrix = 96 × 96, axial slices, and b-value = 1000 s/mm^2^. Further details on MRI acquisition parameters and preprocessing procedures are described in dataset article [45].

Neuroimaging data analysis was conducted using the CONN functional connectivity toolbox version 22.407 [46]. Preprocessing of native images included the following steps: realignment, susceptibility distortion correction, slice timing correction, co-registration, and normalization, followed by functional smoothing. After preprocessing, the compiled images underwent two stages of denoizing. The first stage involved linear regression of potential noise signals in the global BOLD contrast using the “aCompCor” method. The second stage involved applying a temporal frequency filter, removing frequencies below 0.008 Hz or above 0.09 Hz to retain only low-frequency oscillations, thereby minimizing the effects of head motion and other noise sources.

Seed-based connectivity maps were generated to characterize patterns of functional connectivity with 164 «HPC-ICA networks and Harvard-Oxford atlas ROIs» [47]. Functional connectivity strength was quantified using Fisher-transformed bivariate correlation coefficients derived from a weighted general linear model [46]. These coefficients were calculated for each seed-target pair, modeling associations between their respective blood oxygenation level dependence signal time series. To mitigate transient magnetization effects at the start of each scan, individual runs were weighted using a step function convolved with a statistical parametric mapping canonical hemodynamic response function, followed by rectification.

Group-level analyses employed a general linear model. For each voxel, a separate general linear model was estimated, with first-level connectivity measures as dependent variables and group or subject-level identifiers as independent variables. Hypotheses were evaluated using multivariate parametric statistics with random-effects across subjects and sample covariance estimation across measurements. Statistical inferences were performed at the cluster level, examining contiguous voxel groups. Cluster-level significance was assessed using parametric statistics based on Gaussian random field theory, with thresholds set at *p*  < 0.001 for voxel-level significance and p-FWE/p-FDR < 0.05 for familywise error- or false discovery rate-corrected cluster sizes [48].

To evaluate differences in functional connectivity between the control and study groups, based on temperament traits, a one-way ANCOVA with covariate interaction was employed, enabling regression comparison between groups. Seeds included key regions of interest from four large-scale brain networks: DMN: medial prefrontal cortex, bilateral lateral parietal cortex, posterior cingulate cortex; SN: anterior cingulate cortex, bilateral anterior insular cortex, bilateral supramarginal gyrus; FPN: bilateral lateral prefrontal cortex, bilateral posterior parietal cortex; SomatoSensory Network: regions involved in somatosensory processing.

Psychometric data were analyzed using Jamovi software [49]. Descriptive statistics, normality testing (Shapiro–Wilk test), and homogeneity of variance testing (Levene's test) were conducted. Parametric data were analyzed using Student's *t*-test (Welch's *t*-test for unequal variances), one-way ANOVA (Fisher's F-test; Welch's F-test for unequal variances), and post hoc comparisons via Tukey's test (Games-Howell for unequal variances). Correlations were assessed using Pearson's correlation coefficient. Nonparametric data were analyzed with Mann–Whitney *U* test, Kruskal–Wallis one-way ANOVA, and pairwise comparisons using Dwass-Steel–Critchlow–Fligner tests. Results are presented as measures of central tendency: Mean and standard deviation M(SD) for parametric data; median and interquartile range Me[IQR] for non-parametric data; and percentage (%) or absolute frequency (n) for categorical variables.

## 3. Results

The comparative analysis of temperamental features in BD and HC revealed significant differences in all four traits. Thus, patients with BD demonstrated higher levels of Harm Avoidance (t-Welch's (82.8) = 4.85; *p* < 0.001) and Novelty Seeking (t-Welch's (87.3) = 4.37; *p* < 0.001). Conversely, Reward Dependence (t-Welch's (87.1) = −2.50; *p*=0.014) and Persistence (*U* = 998; *p*=0.002) were significantly lower compared to the temperamental characteristics of HC (Table 1).

**Figure 1:**
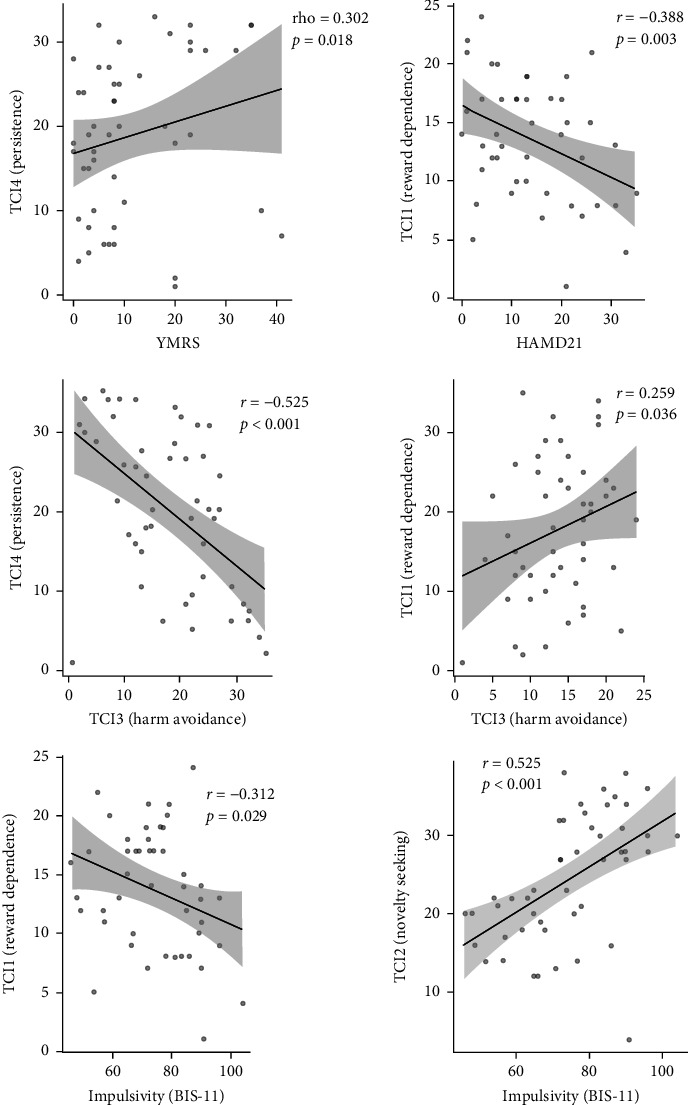
Correlation matrices of affective symptom severity, impulsivity and temperament traits. Abbreviations: HAMD21, Hamilton Depression Rating Scale (21 items); p, level of statistical significance; r, Pearson's correlation coefficient; rho, Spearman's correlation coefficient; YMRS, Young Mania Rating Scale.

Higher Persistence in BD was associated with greater severity of manic symptoms (Spearman's rho = 0.302, *p*=0.018), while lower Reward Dependence was associated with greater severity of depressive symptoms (Pearson's *r* = −0.388, *p*=0.003). When assessing the internal correlation between temperament traits in patients with BD, we found that Harm Avoidance negatively correlates with Persistence (Pearson's *r* = −0.525, *p* < 0.001) and positively with Reward Dependence (Pearson's *r* = 0.259, *p*=0.036) (Figure 1).

### 3.1. Neuroimaging

**Figure 2:**
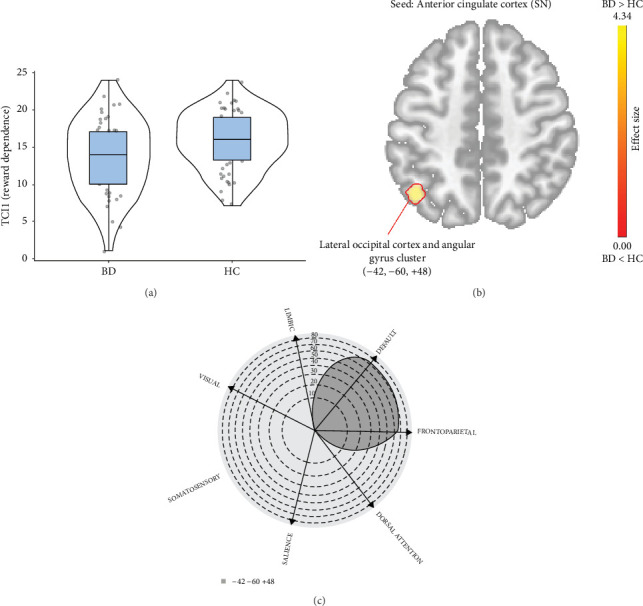
Functional connectivity alteration due to lower Reward Dependence in BD patients compared to the HC. (A) Violin plots showing the difference in the severity of the Reward Dependence (t-Welch's = 0.486; *p*=0.014); (B) Seed-based connectivity map for the anterior cingulate cortex seed (SN), demonstrating the altered functional connectivity in BD compared to HC based on the severity of the Reward Dependence; (C) Polar display showing the number of altered voxels (shown in B) in each large-scale brain network.

The lower Reward Dependence in patients with BD, compared to the HC, was associated with increased connectivity of the anterior cingulate cortex (SN) with a cluster of structures in the left primary visual cortex (147 voxels with a center at −42, −60, +48; p-FDR < 0.05). A larger number of voxels with altered functional connectivity were located in the DMN and FPN (Figure 2).

**Figure 3:**
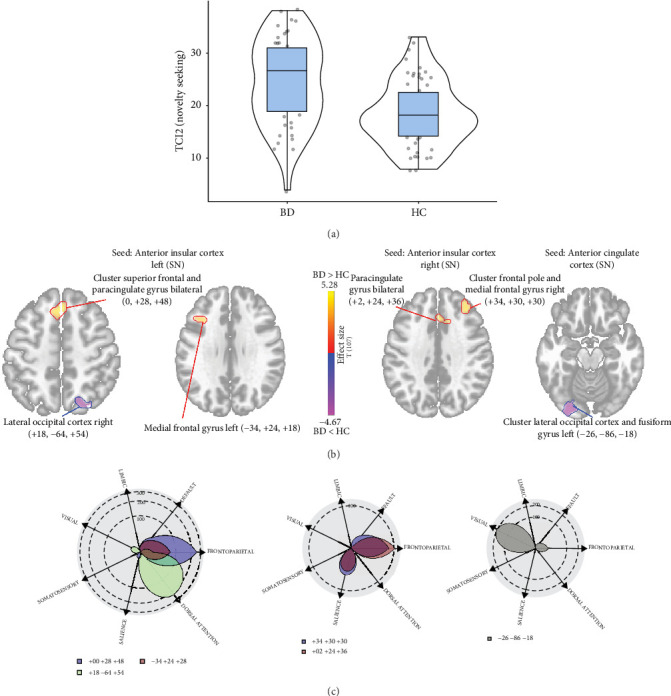
Functional connectivity alteration due to higher Novelty Seeking in BD patients compared to the HC. (A) Violin plots showing the difference in the severity of the temperament trait “Novelty Seeking” (t-Welch's = 0.848; *p* < 0.001); (B) Seed-based connectivity map for the anterior cingulate cortex and anterior insula seeds (SN), demonstrating the difference in their functional connectivity in BD compared to HC based on the severity of the temperament trait “Novelty Seeking”; (C) Polar display showing the number of altered voxels (shown in B) in each network.

An increased level of the Novelty Seeking in BD patients compared to the HC was also associated with changes in the SN. Specifically, it was linked to reduced connectivity of the anterior cingulate cortex with a cluster of structures in the left primary visual cortex (234 voxels centered at −26, −86, −18; p-FWE < 0.01) and reduced functional connectivity between the left anterior insular cortex and the right lateral occipital cortex (260 voxels centered at +18, −64, +54; p-FWE < 0.01). In contrast, increased connectivity was observed between the right anterior insular cortex and the bilateral paracingulate gyrus (172 voxels centered at +2, +24, +36; p-FWE = 0.02) and a cluster in the frontal pole and middle frontal gyrus (205 voxels centered at +34, +30, +30; p-FWE < 0.01). Functional connectivity of the left anterior insular cortex was elevated with a cluster in the superior frontal and paracingulate gyri bilaterally (310 voxels centered at 0, +28, +48; p-FWE < 0.01) and with the left middle frontal gyrus (203 voxels centered at −34, +24, +28; p-FWE < 0.01). At the inter-network level, Novelty Seeking was characterized by increased functional connectivity between SN and FPN and decreased functional connectivity between SN and Visual Network as well as Dorsal Attention Network (Figure 3).

**Figure 4:**
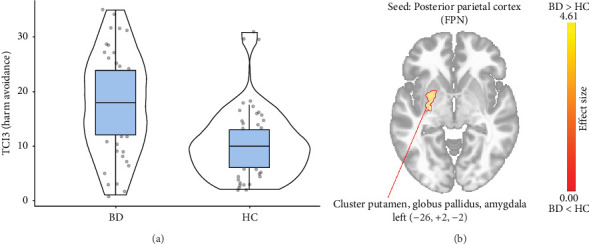
Functional connectivity alterations due to the higher Harm Avoidance level in BD patients compared to the HC. (A) Violin plots showing the difference in the severity of the Harm Avoidance (t-Welch's = 0.945; *p* < 0.001); (B) Seed-based connectivity map for the posterior parietal cortex seed (FPN), demonstrating the difference in its functional connectivity in BD compared to HC based on the severity of the higher Harm Avoidance.

A decreased level of the temperament trait Harm Avoidance in BD patients compared to the HC was associated with enhanced connectivity of the posterior parietal cortex (FPN) with a cluster of subcortical structures, including the putamen, globus pallidus, and amygdala on the left side (287 voxels centered at −26, +2, −2; p-FWE < 0.01) (Figure 4).

**Figure 5:**
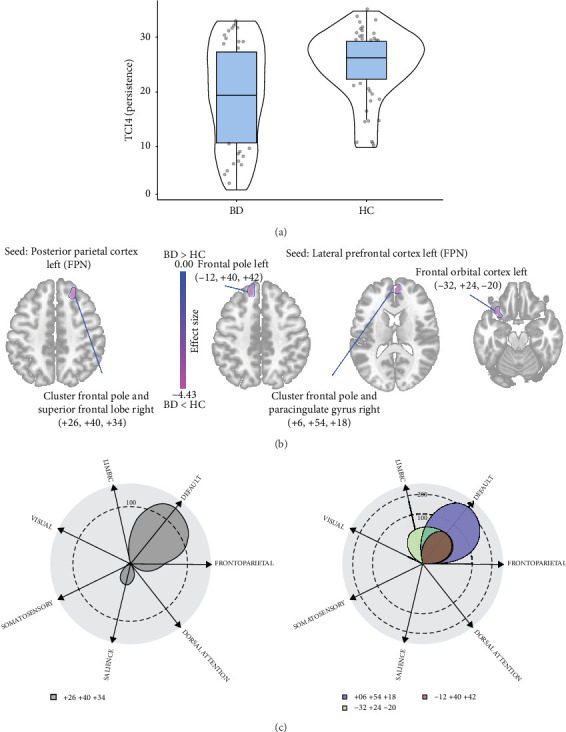
Functional connectivity alterations due to the severity of the Persistence in BD patients compared to the HC. (A) Violin plots showing the difference in the severity of the Persistence (t-Welch's = 0.848; *p* < 0.001); (B) Seed-based connectivity map for the anterior cingulate cortex and anterior insula seeds (SN), demonstrating the difference in their functional connectivity in BD compared to HC based on the severity of the Persistence; (C) Polar display showing the number of altered voxels (shown in B) in each network.

Finally, a lower Persistence in BD patients compared to the HC was also associated with changes in the FPN. This included a decreased functional connectivity of the left posterior parietal cortex with a cluster in the frontal pole and superior frontal gyrus on the right side (214 voxels centered at +26, −40, +34; p-FWE < 0.01); reduced functional connectivity between the left lateral prefrontal cortex and the right orbitofrontal cortex (118 voxels centered at −32, +24, −20; p-FDR = 0.03), as well as with a cluster in the frontal pole and anterior cingulate cortex on the right side (234 voxels centered at +6, +54, +18; p-FWE = 0.02). At the inter-network level, Persistence was primarily characterized by decreased functional connectivity between FPN and DMN (Figure 5).

## 4. Discussion

The results revealed a specific spectrum of temperament trait alterations of patients with BD compared to HC: lower scores on the dimensions of Reward Dependence and Persistence, and higher scores on Novelty Seeking and Harm Avoidance. Neuroimaging data analysis identified alterations in functional connectivity within the SN and FPN, as well as in their interactions with the DMN, Dorsal Attention Network, Visual Network, and basal nuclei, associated with these temperament traits in BD patients compared to HC. The primary role of the SN is its involvement in attentional control through the detection of subjectively salient events and the provision of control signals to the FPN for goal-directed actions or to the DMN for self-referential processes, social cognition, episodic and autobiographical memory, language and semantic memory, and mind-wandering [50–52]. Of particular interest, warranting further investigation, is the striking similarity of the temperament trait alterations observed in our study with those reported in a French cohort of 570 patients with Parkinson's disease [53].

### 4.1. Reward Dependence

Reduced Reward Dependence previously described as characteristic temperament trait in patients with BD [54, 55]. Similarly, this trait is often markedly lower in patients with major depressive disorder [56, 57], particularly in those with treatment-resistant depression [58]. Conversely, elevated Reward Dependence has been associated with a reduced risk of psychotic depression [59]. Longitudinal studies on large samples have shown this trend; however, its statistical significance was not consistently confirmed, possibly due to gender-specific sampling characteristics: a significant increase in Reward Dependence that associated with a reduced risk of psychotic depression was noted exclusively among women in a 23-year follow-up study [60]. It is worth noting that decreased Reward Dependence, alongside increased Harm Avoidance, underlies asociality, which potentially increases the risk of schizophrenia [61], and is associated with a greater likelihood of paranoid ideation in otherwise healthy individuals [62].

Decreased Reward Dependence in BD patients was associated with increased functional connectivity between the SN and the DMN, as well as between the SN and the FPN, a finding consistent with earlier research [63]. Disruption of the anticorrelation between the SN and the DMN [64] has been linked to goal-directed behavioral deficits due to impaired introspection and attentional control [65]. Alterations in network interactions, such as increased connectivity of the SN with the DMN and FPN, likely underlie impairments in emotional processing of external and internal stimuli. These alterations may manifest as difficulties in regulating behavior aimed at achieving long-term goals, explaining the observed neuroimaging changes and the association of this temperament trait with depressive symptoms and impulsivity.

### 4.2. Novelty Seeking

The correlation of higher scores on the Novelty Seeking dimension with impulsivity confirms the association of this temperament trait with impulsive decision-making, rapid loss of self-control, frustration avoidance, and an extravagant perception of rewards linked to dopamine levels [17, 27]. Additionally, an increased number of suicide attempts in patients with affective disorders is associated with elevated Novelty Seeking [66]. The findings align with previous studies suggesting that heightened Novelty Seeking is specific to BD [67, 68], supporting the prevailing hyperdopaminergic theory of BD pathogenesis [69]. The identified changes in functional connectivity associated with Novelty Seeking reflect enhanced connectivity between controlling prefrontal regions and the structures of the SN. Previous research has also demonstrated a strong association between Novelty Seeking and alterations in the SN, which have been linked to personal relevance [70]. This supports the hypothesis that changes in resting-state SN connectivity are related to the behavioral inhibition system's activity, characteristic of this temperament trait [63, 71, 72]. Our results indicate decreased SN connectivity with the visual network and dorsal attention network, alongside increased connectivity with the FPN. This may theoretically reflect a focus in BD patients not on real external and internal stimuli but rather on goal-directed pursuits of new sensations or emotions, often associated with risk-taking behavior.

### 4.3. Harm Avoidance

The personality traits described as “depressive temperament” by E. Kraepelin and “depressive psychopathy” by K. Schneider are largely consistent with today's definition of the Harm Avoidance dimension [73]. Modern studies, along with R. Cloninger's own hypotheses, suggest that individuals with high Harm Avoidance scores are prone to excessive negative reactions to aversive stimuli [74]. Moreover, greater Harm Avoidance is a risk factor for the development of affective disorders [59, 75, 76], is observed in healthy relatives of patients with depression [77], and is associated with an increased risk of suicide attempts [78]. Longitudinal prospective studies have also reported sustained elevations in Harm Avoidance scores among young patients with affective disorders, independently of the disease phase, over 2 years [79]. Patients with affective disorders demonstrate reduced emotional resilience associated with higher Harm Avoidance scores [80]. Finally, meta-analytic data on temperament traits in affective disorders indicate that elevated Harm Avoidance is a temperament marker for all affective disorders [68].

Higher Harm Avoidance scores were associated with increased functional connectivity of the FPN with basal ganglia structures (putamen, globus pallidus, and amygdala). The prefrontal cortex has been identified as the most critical functional brain region for the Harm Avoidance dimension [41]. Reduced binding potential in the orbitofrontal cortex negatively correlates with Harm Avoidance in patients with depression [81], leading to executive dysfunction in these patients, who exhibit high Harm Avoidance [82]. The severity of Harm Avoidance and altered prefrontal-subcortical connectivity are linked to manifestations of anxiety and fear extinction responses [83–86]. Previous studies have also suggested that changes in this connectivity underlie inhibited behavior in patients with higher Harm Avoidance scores [72]. The findings may indicate reduced higher-order control and impaired top-down regulation of the FPN in favor of increased basal ganglia activation. This possibly determines clinical manifestations such as heightened affective responses to aversive stimuli, increased anxiety, and reduced executive function efficiency.

### 4.4. Persistence

Patients with affective disorders, mostly with depression, often exhibit lower levels of Persistence what associated with reduced emotional stability [80, 87, 88]. Moreover, greater Persistence is correlated with better overall health and happiness, but only when coupled with higher frustration tolerance, i.e., lower Harm Avoidance [89]. On the other hand, lower Persistence could not be a trait marker of BD [67]. Moreover, a 15-year study involving 2212 respondents found that a higher polygenic risk for depression predicted higher Persistence from early adulthood to middle age [90]. In our sample, the higher temperament trait of Persistence was associated with higher manic symptoms and lower Harm Avoidance.

Reduced functional connectivity between the structures of the FPN and DMN was linked to lower Persistence scores. Altered DMN–FPN functional connectivity is thought to underlie deficits in processing, managing, and regulating affective stimuli [91]. It could also be explained by impairment of networks switching according to the long-term goal directed behavior [92]. Cognitive impairments in BD also appear to be associated with aberrant connectivity within the DMN and FPN [93]. The most plausible explanation for the observed changes in frontal regions, which combine a positive correlation between persistence and manic symptoms with a strong negative correlation between Persistence and Harm Avoidance, seems to be reduced cognitive control in goal-related behavior. This also may lead to more decisive and reckless behavior in BD patients, reflecting diminished behavioral persistence in uncertain decision-making scenarios [42, 94].

## 5. Limitations

This study has several limitations. First of all, the study's cross-sectional design precludes causal inferences regarding the relationship between temperamental traits and functional connectivity alterations. Longitudinal studies are needed to explore the temporal dynamics of these associations and their potential role in the progression of BD. As well as the sample size may limit the generalizability of the findings to broader BD populations. A larger and more diverse sample, including participants from different age groups, cultural backgrounds, and phases of the disorder, could enhance the robustness of the results. Temperamental traits and impulsivity were evaluated using self-report measures, which may be subject to response biases, such as social desirability or recall bias. We did not control the effect of psychotropic medications and comorbidities which could also influence brain connectivity. Despite these limitations, the findings provide important insights into the neural correlates of temperamental traits in BD and lay the groundwork for future research.

## 6. Conclusions

We identified some specific temperament traits related in the pathophysiological processes of BD. It reflects in functional connectivity alterations within the SN and FPN and their interactions with the DMN, Dorsal Attention Network, Visual Network, and basal nuclei, closely linked to clinical symptoms severity. The association between increased impulsivity and temperament traits, which are critical for its development and expression, underscores the importance of impulsivity in the structure of BD. These findings highlight the need for assessing crossdiagnostic phenomena to improve diagnostic accuracy.

## Figures and Tables

**Table 1:** Demographic data and comparison of key temperamental characteristics based on TCI scale results.

Variables	Bipolar disorder (*n* = 49)	Healthy controls (*n* = 49)	Effect size	*p*
Age, years (M(SD))	35,3 (9,0)	32,6 (8,6)	0,750	0,062
Gender (female/male)	28/21	27/22	0,151	0,697
Reward dependence (M(SD))	13,6 (5,1)	15,8 (3,8)	0,486	0,014
Novelty seeking (M(SD))	24,4 (8,0)	18,4 (6,1)	0,848	<0,001
Harm avoidance (M(SD))	17,8 (9,0)	10,4 (6,3)	0,945	<0,001
Persistence (Me[IQR])	19,0 [17,0]	26,0 [7,00]	0,343	0,002

## Data Availability

The data that support the findings of this study are available from the corresponding author upon reasonable request.

## References

[1] Grande I., Berk M., Birmaher B., Vieta E. (2016). Bipolar Disorder. *The Lancet*.

[2] GBD 2019 Diseases and Injuries Collaborators (2020). Global Burden of 369 Diseases and Injuries in 204 Countries and Territories, 1990–2019: A Systematic Analysis for the Global Burden of Disease Study 2019. *The Lancet*.

[3] Barnett J. H., Smoller J. W. (2009). The Genetics of Bipolar Disorder. *Neuroscience*.

[4] Kasyanov E. D., Merkulova T. V., Kibitov A. O., Mazo G. E. (2020). Genetics of Bipolar Spectrum Disorders: Focus on Family Studies Using Whole Exome Sequencing. *Russian Journal of Genetics*.

[5] Lai J., Li S., Wei C. (2024). Mapping the Global, Regional and National Burden of Bipolar Disorder From 1990 to 2019: Trend Analysis on the Global Burden of Disease Study 2019. *The British Journal of Psychiatry*.

[6] Zhong Y., Chen Y., Su X. (2024). Global, Regional and National Burdens of Bipolar Disorders in Adolescents and Young Adults: A Trend Analysis From 1990 to 2019. *General Psychiatry*.

[7] Daveney J., Panagioti M., Waheed W., Esmail A. (2019). Unrecognized Bipolar Disorder in Patients With Depression Managed in Primary Care: A Systematic Review and Meta-Analysis. *General Hospital Psychiatry*.

[8] Mosolov S. N., Ushkalova A. V., Kostyukova E. G., Shafarenko A. A., Alfimov P. V., Kostyukova A. B. (2014). Diagnostics of Bipolar II Disorder Among Patients With the Current Diagnosis of Recurrent Depressive Disorder. *Sovrem. ter. psih. Rasstrojstv [Сurrent Therapy of Mental Disorders]*.

[9] Shen H., Zhang L., Xu C., Zhu J., Chen M., Fang Y. (2018). Analysis of Misdiagnosis of Bipolar Disorder in An Outpatient Setting. *Shanghai Archives of Psychiatry*.

[10] Petrova N. N. (2024). Diagnosis and Treatment of Bipolar Disorder. *Sovrem. ter. psih. Rasstrojstv [Сurrent Therapy of Mental Disorders]*.

[11] Maes M. H. J., Stoyanov D. (2022). False Dogmas in Mood Disorders Research: Towards a Nomothetic Network Approach. *World Journal of Psychiatry*.

[12] Malhi G. S., Irwin L., Hamilton A. (2018). Modelling Mood Disorders: An ACE Solution?. *Bipolar Disorders*.

[13] Malhi G. S., Bell E., Bassett D. (2021). The 2020 Royal Australian and New Zealand College of Psychiatrists Clinical Practice Guidelines for Mood Disorders. *The Australian and New Zealand Journal of Psychiatry*.

[14] Whitton A. E., Pizzagalli D. A. (2022). Anhedonia in Depression and Bipolar Disorder. *Current Topics in Behavioral Neurosciences*.

[15] Markin K. V., Shamrey V. K., Tarumov D. A. (2023). Role of Impulsivity and Its Types in the Structure of Bipolar Affective Disorder. *Bulletin of Medical Science*.

[16] Morozova M. A., Potanin S. S., Burminskiy D. S., Rupchev G. E., Beniashvili A. G., Lepilkina T. A. (2022). Impulsivity in Bipolar Affective Disorder: Prevalence and Comorbidity. *Psychiatry (Moscow) [Psikhiatriya]*.

[17] Ovchinnikov A. V., Vazagaeva T. I., Akhapkin R. V., Volel B. A. (2023). Predictive Capabilities of the Cloninger Temperament and Character Inventory (TCI) in Evaluating the Effectiveness of Antidepressant Pharmacotherapy. Systematic Review and Meta-Analysis. *Neurology, Neuropsychiatry, Psychosomatics*.

[18] Garcia D., Kazemitabar M., Stoyanova K., Stoyanov D., Cloninger C. R. (2022). Differences in Subjective Well-Being Between Individuals With Distinct Joint Personality (Temperament-Character) Networks in a Bulgarian Sample. *PeerJ*.

[19] Simonetti A., Luciano M., Sampogna G. (2023). Effect of Affective Temperament on Illness Characteristics of Subjects With Bipolar Disorder and Major Depressive Disorder. *Journal of Affective Disorders*.

[20] Karam E. G., Saab D., Jabbour S., Karam G. E., Hantouche E., Angst J. (2023). The Role of Affective Temperaments in Bipolar Disorder: The Solid Role of the Cyclothymic, the Contentious Role of the Hyperthymic, and the Neglected Role of the Irritable Temperaments. *European Psychiatry*.

[21] Perugi G., Toni C., Maremmani I. (2012). The Influence of Affective Temperaments and Psychopathological Traits on the Definition of Bipolar Disorder Subtypes: A Study on Bipolar I Italian National Sample. *Journal of Affective Disorders*.

[22] Qiu F., Akiskal H. S., Kelsoe J. R., Greenwood T. A. (2017). Factor Analysis of Temperament and Personality Traits in Bipolar Patients: Correlates With Comorbidity and Disorder Severity. *Journal of Affective Disorders*.

[23] Fountoulakis K. N., Gonda X., Koufaki I., Hyphantis T., Cloninger C. R. (2016). The Role of Temperament in the Etiopathogenesis of Bipolar Spectrum Illness. *Harvard Review of Psychiatry*.

[24] Higier R. G., Jimenez A. M., Hultman C. M. (2014). Enhanced Neurocognitive Functioning and Positive Temperament in Twins Discordant for Bipolar Disorder. *American Journal of Psychiatry*.

[25] Cloninger C. R. (1987). A Systematic Method for Clinical Description and Classification of Personality Variants. A Proposal. *Archives of General Psychiatry*.

[26] Cloninger C. R., Cloninger K. M., Zwir I., Keltikangas-Järvinen L. (2019). The Complex Genetics and Biology of Human Temperament: A Review of Traditional Concepts in Relation to New Molecular Findings. *Translational Psychiatry*.

[27] Cloninger C. R., Svrakic D. M., Przybeck T. R. (1993). A Psychobiological Model of Temperament and Character. *Archives of General Psychiatry*.

[28] Gencheva T. M., Valkov B. V., Kandilarova S. S., Maes M. H. J., Stoyanov D. S. (2024). Diagnostic Value of Structural, Functional and Effective Connectivity in Bipolar Disorder. *Acta Psychiatrica Scandinavica*.

[29] Brenner A. M., Claudino F. C. de A., Burin L. M. (2022). Structural Magnetic Resonance Imaging Findings in Severe Mental Disorders Adult Inpatients: A Systematic Review. *Psychiatry Research: Neuroimaging*.

[30] Claeys E. H. I., Mantingh T., Morrens M., Yalin N., Stokes P. R. A. (2022). Resting-State fMRI in Depressive and (hypo)manic Mood States in Bipolar Disorders: A Systematic Review. *Progress in Neuro-Psychopharmacology and Biological Psychiatry*.

[31] Schumer M. C., Chase H. W., Rozovsky R., Eickhoff S. B., Phillips M. L. (2023). Prefrontal, Parietal, and Limbic Condition-Dependent Differences in Bipolar Disorder: A Large-Scale Meta-Analysis of Functional Neuroimaging Studies. *Molecular Psychiatry*.

[32] Markin K. V. (2023). Resting-State Functional Magnetic Resonance Imaging in Bipolar Affective Disorder. A Systematized Umbrella Review. *V.M. Bekhterev Review of Psychiatry and Medical Psychology*.

[33] Martino M., Magioncalda P. (2022). Tracing the Psychopathology of Bipolar Disorder to the Functional Architecture of Intrinsic Brain Activity and Its Neurotransmitter Modulation: A Three-Dimensional Model. *Molecular Psychiatry*.

[34] Han K.-M., De Berardis D., Fornaro M., Kim Y.-K. (2019). Differentiating Between Bipolar and Unipolar Depression in Functional and Structural MRI Studies. *Progress in Neuro-Psychopharmacology and Biological Psychiatry*.

[35] Nunes A., Schnack H. G., Ching C. R. K. (2020). Using Structural MRI to Identify Bipolar Disorders – 13 Site Machine Learning Study in 3020 Individuals From the ENIGMA Bipolar Disorders Working Group. *Molecular Psychiatry*.

[36] Huth F., Tozzi L., Marxen M. (2023). Machine Learning Prediction of Estimated Risk for Bipolar Disorders Using Hippocampal Subfield and Amygdala Nuclei Volumes. *Brain Sciences*.

[37] Mikolas P., Marxen M., Riedel P. (2024). Prediction of Estimated Risk for Bipolar Disorder Using Machine Learning and Structural MRI Features. *Psychological Medicine*.

[38] Dominicus L. S., van Rijn L., van der A A. (2023). fMRI Connectivity as a Biomarker of Antipsychotic Treatment Response: A Systematic Review. *NeuroImage: Clinical*.

[39] Salman M. S., Verner E., Bockholt H. J. (2023). Multi-Study Evaluation of Neuroimaging-Based Prediction of Medication Class in Mood Disorders. *Psychiatry Research: Neuroimaging*.

[40] Seitzman B. A., Gratton C., Laumann T. O. (2019). Trait-Like Variants in Human Functional Brain Networks. *Proceedings of the National Academy of Sciences*.

[41] Zhong S., Lin J., Zhang L. (2024). Neural Correlates of Harm Avoidance: A Multimodal Meta-Analysis of Brain Structural and Resting-State Functional Neuroimaging Studies. *Translational Psychiatry*.

[42] Jiang R., Calhoun V. D., Zuo N. (2018). Connectome-Based Individualized Prediction of Temperament Trait Scores. *NeuroImage*.

[43] Zwir I., Arnedo J., Mesa A., del Val C., de Erausquin G. A., Cloninger C. R. (2023). Temperament & Character Account for Brain Functional Connectivity at Rest: A Diathesis-Stress Model of Functional Dysregulation in Psychosis. *Molecular Psychiatry*.

[44] Poldrack R. A., Congdon E., Triplett W. (2016). A Phenome-Wide Examination of Neural and Cognitive Function. *Scientific Data*.

[45] Gorgolewski K. J., Durnez J., Poldrack R. A. (2017). Preprocessed Consortium for Neuropsychiatric Phenomics Dataset. *F1000Research*.

[46] Whitfield-Gabrieli S., Nieto-Castanon A. (2012). Conn: A Functional Connectivity Toolbox for Correlated and Anticorrelated Brain Networks. *Brain Connectivity*.

[47] Desikan R. S., Ségonne F., Fischl B. (2006). An Automated Labeling System for Subdividing the Human Cerebral Cortex on MRI Scans into Gyral Based Regions of Interest. *NeuroImage*.

[48] Chumbley J., Worsley K., Flandin G., Friston K. (2010). Topological FDR for Neuroimaging. *NeuroImage*.

[49] about—Jamovi. (n.d.) (2024). https://www.jamovi.org/about.html.

[50] Menon V. (2023). 20 Years of the Default Mode Network: A Review and Synthesis. *Neuron*.

[51] Menon V. (2011). Large-Scale Brain Networks and Psychopathology: A Unifying Triple Network Model. *Trends in Cognitive Sciences*.

[52] Uddin L. Q. (2015). Salience Processing and Insular Cortical Function and Dysfunction. *Nature Reviews Neuroscience*.

[53] Boussac M., Arbus C., Colin O. (2022). Personality Assessment With Temperament and Character Inventory in Parkinson’s Disease. *Parkinsonism & Related Disorders*.

[54] Engström C., Brändström S., Sigvardsson S., Cloninger R., Nylander P.-O. (2004). Bipolar Disorder: I. Temperament and Character. *Journal of Affective Disorders*.

[55] Fayyazi Bordbar M. R., Faridhosseini F., Kaviani H., Kazemian M., Samari A. A., Kashani Lotfabadi M. (2014). [Temperament and Character Personality Dimensions in Patients With Bipolar I Disorder]. *Turk Psikiyatri Dergisi = Turkish Journal of Psychiatry*.

[56] Jylhä P., Mantere O., Melartin T. (2011). Differences in Temperament and Character Dimensions in Patients With Bipolar I or II or Major Depressive Disorder and General Population Subjects. *Psychological Medicine*.

[57] Oh H. S., Cloninger C. R. (2024). The Role of Temperament and Character in the Anxiety-Depression Spectrum Among Korean Adults. *Journal of Affective Disorders*.

[58] Takahashi M., Shirayama Y., Muneoka K., Suzuki M., Sato K., Hashimoto K. (2013). Personality Traits as Risk Factors for Treatment-Resistant Depression. *PLoS ONE*.

[59] Ahola A., Rautio N., Timonen M., Nordström T., Jääskeläinen E., Miettunen J. (2023). Premorbid Temperament as Predictor of Onset of Depression: 23-Year Follow-up. *Comprehensive Psychiatry*.

[60] Josefsson K., Merjonen P., Jokela M., Pulkki-Råback L., Keltikangas-Järvinen L. (2011). Personality Profiles Identify Depressive Symptoms Over Ten Years? A Population-Based Study. *Depression Research and Treatment*.

[61] Smith M. J., Cloninger C. R., Harms M. P., Csernansky J. G. (2008). Temperament and Character as Schizophrenia-Related Endophenotypes in Non-Psychotic Siblings. *Schizophrenia Research*.

[62] Saarinen A., Rosenström T., Hintsanen M. (2018). Longitudinal Associations of Temperament and Character With Paranoid Ideation: A Population-Based Study. *Psychiatry Research*.

[63] Li S., Demenescu L. R., Sweeney-Reed C. M., Krause A. L., Metzger C. D., Walter M. (2017). Novelty Seeking and Reward Dependence-Related Large-Scale Brain Networks Functional Connectivity Variation During Salience Expectancy. *Human Brain Mapping*.

[64] Zhao L., Bo Q., Zhang Z., Li F., Zhou Y., Wang C. (2024). Disrupted Default Mode Network Connectivity in Bipolar Disorder: A Resting-State fMRI Study. *BMC Psychiatry*.

[65] Lopez-Larson M. P., Shah L. M., Weeks H. R. (2017). Abnormal Functional Connectivity Between Default and Salience Networks in Pediatric Bipolar Disorder. *Biological Psychiatry: Cognitive Neuroscience and Neuroimaging*.

[66] Liu S.-I., Huang Y.-H., Wu Y.-H. (2017). Temperament Traits in Suicidal and Non-Suicidal Mood Disorder Patients in Taiwan. *Psychiatry Research*.

[67] Nery F. G., Hatch J. P., Glahn D. C. (2008). Temperament and Character Traits in Patients With Bipolar Disorder and Associations With Comorbid Alcoholism or Anxiety Disorders. *Journal of Psychiatric Research*.

[68] Zaninotto L., Solmi M., Toffanin T., Veronese N., Cloninger C. R., Correll C. U. (2016). A Meta-Analysis of Temperament and Character Dimensions in Patients With Mood Disorders: Comparison to Healthy Controls and Unaffected Siblings. *Journal of Affective Disorders*.

[69] Ashok A. H., Marques T. R., Jauhar S. (2017). The Dopamine Hypothesis of Bipolar Affective Disorder: The State of the Art and Implications for Treatment. *Molecular Psychiatry*.

[70] Enzi B., de Greck M., Prösch U., Tempelmann C., Northoff G. (2009). Is Our Self Nothing but Reward? Neuronal Overlap and Distinction Between Reward and Personal Relevance and Its Relation to Human Personality. *PLoS ONE*.

[71] Alloy L. B., Abramson L. Y., Walshaw P. D. (2008). Behavioral Approach System and Behavioral Inhibition System Sensitivities and Bipolar Spectrum Disorders: Prospective Prediction of Bipolar Mood Episodes. *Bipolar Disorders*.

[72] Kyeong S., Kim E., Park H.-J., Hwang D.-U. (2014). Functional Network Organizations of Two Contrasting Temperament Groups in Dimensions of Novelty Seeking and Harm Avoidance. *Brain Research*.

[73] Garanyan N. G. (2009). Depression and Personality: A Review of Foreign Research. Part 1. *Social and Clinical Psychiatry*.

[74] Cloninger C. R., Svrakic D. M., Przybeck T. R. (2006). Can Personality Assessment Predict Future Depression? A Twelve-Month Follow-up of 631 Subjects. *Journal of Affective Disorders*.

[75] Harley J. A., Wells J. E., Frampton C. M. A., Joyce P. R. (2011). Bipolar Disorder and the TCI: Higher Self-Transcendence in Bipolar Disorder Compared to Major Depression. *Depression Research and Treatment*.

[76] Loftus S. T., Garno J. L., Jaeger J., Malhotra A. K. (2008). Temperament and Character Dimensions in Bipolar I Disorder: A Comparison to Healthy Controls. *Journal of Psychiatric Research*.

[77] Farmer A., Mahmood A., Redman K., Harris T., Sadler S., McGuffin P. (2003). A Sib-Pair Study of the Temperament and Character Inventory Scales in Major Depression. *Archives of General Psychiatry*.

[78] Özdemir Yılmaz S., Ertekin Yazıcı A., Yılmaz H. (2024). Association of Temperament and Character Traits With Suicide Probability, Suicide Attempts, and Perceived Stress Level in Patients With Bipolar Disorder. *Behavioral Sciences*.

[79] Rajewska-Rager A., Staniek M., Kucharska-Kowalczyk K. (2022). Temperament and Character Dimensions as Psychological Markers of Mood Disorders in Polish Adolescents and Young Adults—A Prospective Study. *Early Intervention in Psychiatry*.

[80] Tsigkaropoulou E., Michopoulos I., Porichi E., Dafnas K., Serretti A., Ferentinos P. (2024). Temperament and Character Dimensions Explain Self-Reported Resilience Deficits in Patients With Affective Disorders. *International Clinical Psychopharmacology*.

[81] van Heeringen C., Audenaert K., Van Laere K. (2003). Prefrontal 5-HT2a Receptor Binding Index, Hopelessness and Personality Characteristics in Attempted Suicide. *Journal of Affective Disorders*.

[82] Aznar S., Hervig M. E.-S. (2016). The 5-HT2A Serotonin Receptor in Executive Function: Implications for Neuropsychiatric and Neurodegenerative Diseases. *Neuroscience & Biobehavioral Reviews*.

[83] Adhikari A., Lerner T. N., Finkelstein J. (2015). Basomedial Amygdala Mediates Top-Down Control of Anxiety and Fear. *Nature*.

[84] Baeken C., Marinazzo D., Van Schuerbeek P. (2014). Left and Right Amygdala—Mediofrontal Cortical Functional Connectivity Is Differentially Modulated by Harm Avoidance. *PLoS ONE*.

[85] Markett S., Weber B., Voigt G. (2013). Intrinsic Connectivity Networks and Personality: The Temperament Dimension Harm Avoidance Moderates Functional Connectivity in the Resting Brain. *Neuroscience*.

[86] Sotres-Bayon F., Bush D. E. A., LeDoux J. E. (2004). Emotional Perseveration: An Update on Prefrontal-Amygdala Interactions in Fear Extinction. *Learning & Memory*.

[87] Osher Y., Lefkifker E., Kotler M. (1999). Low Persistence in Euthymic Manic-Depressive Patients: A Replication. *Journal of Affective Disorders*.

[88] Balestri M., Porcelli S., Souery D. (2019). Temperament and Character Influence on Depression Treatment Outcome. *Journal of Affective Disorders*.

[89] Cloninger C. R., Zohar A. H., Hirschmann S., Dahan D. (2012). The Psychological Costs and Benefits of Being Highly Persistent: Personality Profiles Distinguish Mood Disorders From Anxiety Disorders. *Journal of Affective Disorders*.

[90] Lavonius V., Keltikangas-Järvinen L., Hamal Mishra B. (2024). Polygenic Risk for Depression Predicting Temperament Trajectories Over 15 Years—A General Population Study. *Journal of Affective Disorders*.

[91] Rai S., Griffiths K. R., Breukelaar I. A. (2021). Default-Mode and Fronto-Parietal Network Connectivity During Rest Distinguishes Asymptomatic Patients With Bipolar Disorder and Major Depressive Disorder. *Translational Psychiatry*.

[92] Menon V., D’Esposito M. (2022). The Role of PFC Networks in Cognitive Control and Executive Function. *Neuropsychopharmacology*.

[93] Fortea L., Ysbaek-Nielsen A. T., Macoveanu J. (2023). Aberrant Resting-State Functional Connectivity Underlies Cognitive and Functional Impairments in Remitted Patients With Bipolar Disorder. *Acta Psychiatrica Scandinavica*.

[94] Gusnard D. A., Ollinger J. M., Shulman G. L. (2003). Persistence and Brain Circuitry. *Proceedings of the National Academy of Sciences*.

